# Relation between Oral Health Status and Electrocardiogram ST Segment Changes in a Group of Patients with Myocardial Infarction

**DOI:** 10.5681/joddd.2013.027

**Published:** 2013-08-30

**Authors:** Atousa Aminzadeh, Masoud Ahmadi, Sayyed Mohsen Hosseini

**Affiliations:** ^1^Assistant Professor, Department of Oral Pathology, Khorasgan (Isfahan) Branch, Islamic Azad University, Isfahan, Iran; ^2^Student of Dentistry, School of Dentistry, Khorasgan (Isfahan) Branch, Islamic Azad University, Isfahan, Iran; ^3^Associate Professor, Department of Epidemiology, Isfahan University of Medical Sciences, Isfahan, Iran

**Keywords:** DMFT index, electrocardiogram, oral health, periodontal index, ST segment

## Abstract

***Background and aims.*** Only half to two-thirds of cardiovascular diseases can be explained by the classic risk factors. It is believed that chronic oral inflammation is a potent risk factor for systemic diseases. Studies show that electrocardiogram ST segment changes can be predictive of myocardial infarction outcome. In this study the relation between electrocardio-gram ST segment changes and oral health is evaluated.

***Materials and methods.*** In this cross-sectional study, thirty-six patients (14 females and 22 males) with myocardial infarction were enrolled. Oral health indices including DMFT index, probing depth, clinical attachment loss and bleeding on probing were recorded for each patient. DMFT index, PD, CAL as continuous variables and BOP as a categorical variable were compared with ST segment changes by independent t-test and chi-squared test, respectively (α=0.05).

***Results.*** DMFT index, BOP and PD revealed no statistically significant relation with ST segment groups. CAL showed a statistically significant difference within ST segment groups (P=0.003, OR=1.68).

***Conclusion.*** Clinical attachment loss was significantly higher in patients with ST segment depression, while no correla-tion was seen between probing depth, bleeding on probing and DMFT index with ST segment elevation or depression.

## Introduction

WHO defines oral health as "a state of being free from chronic mouth and facial pain, oral and throat cancer, oral sores, birth defects such as cleft lip and palate, periodontal (gum) disease, tooth decay and tooth loss, and other diseases and disorders that affect the oral cavity."^1^ It is believed that chronic oral inflammation is an effective risk factor for some systemic diseases such as cerebro- and cardiovascular diseases, diabetes mellitus, respiratory disease and low birth weight.^2-5^ There is increasing evidence that inflammatory events may contribute to atherogenesis and cardiovascular disease.^6^

**Figure 1:**
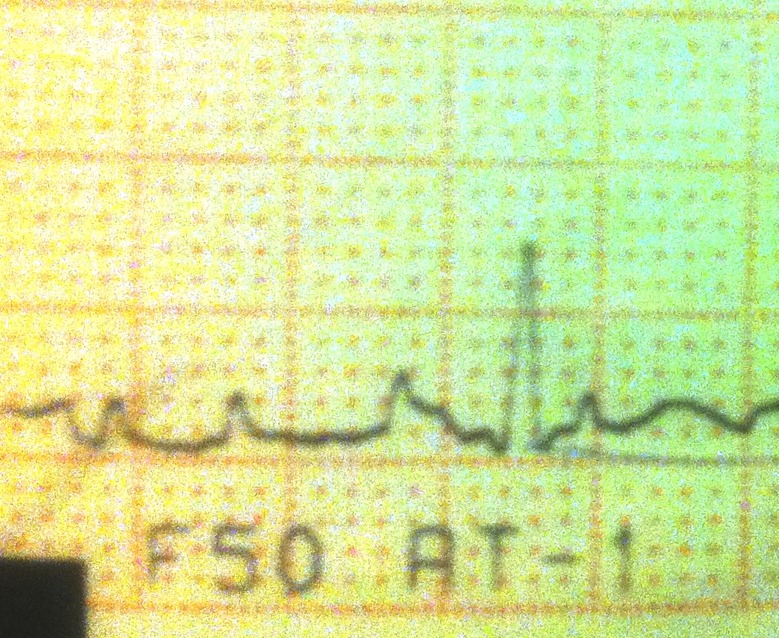
ST elevation (↓) in relation to isoelectric line is shown ( ).

**Figure 2:**
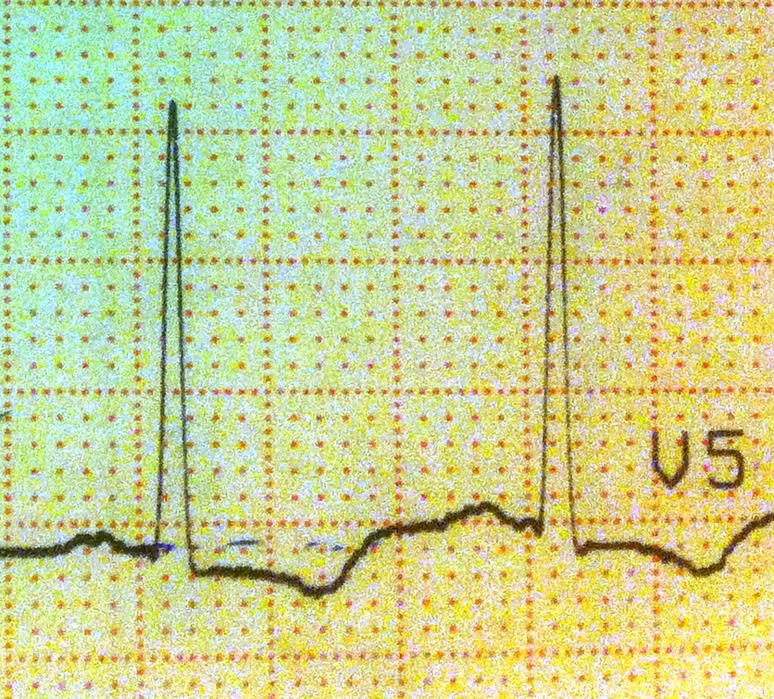
ST depression (↑) in relation to isoelectric line is shown ( ).

Electrocardiogram is a graphic recording of the electrical potential produced by heart muscles. ST segment is usually iso-electric and its depression or elevation is determined in comparison to the iso-electric line(Figures 1 and 2).^7^

Adler et al suggested that ST segment elevation can be a predictive factor for poor outcome in patients with acute myocardial infarction.^8^ Barabess et al believe ST segment depression might be a prognostic feature in AMI patients without ST elevation.^9^ In this study the aim was to investigate the relationship between oral health and electrocardiogram ST segment changes to evaluate whether oral health would be related to the given predictive factors (ST segment changes) in prognosis of myocardial infarction.

## Materials and Methods

Thirty-six patients over 42 hospitalized with diagnosis of myocardial infarction in Isfahan, January-June 2011, were enrolled in the study. Edentulous patients and patients with a history of infection in the past 6 months, systemic diseases and patients receiving periodontal treatment in the past 6 months were excluded from study. According to the electrocardiogram taken on the first day of entry patients were divided to two groups: ST segment elevation (study group A: n=23) and St segment depression (study group B: n=13).

The groups were adjusted for age, gender, smoking habits, regular exercise and family history of cardiac diseases. Electrocardiograms were taken using the same device (Schiller Cardiovitat-1) and interpreted by one specialist. Oral health was assessed in both groups by one educated researcher by means of DMFT index (decayed, missing, filled teeth) and periodontal indices: probing depth (PD), clinical attachment loss (CAL) and bleeding on probing (BOP). Instruments used to analyze oral health status were Michigan O probe with Williams marking. Data gathered were analyzed by the use of t-test and chi-square statistical tests.

## Results

Mean age of the patients (14 females and 22 males) was 60.56 ±10.90 years. BOP was seen in 34.8% of cases in

group A and 53.8% in group B. As shown in Table 1, BOP did not reveal any significant differences between groups A and B (P=0.22) (Table 1).

**Table 1:** Comparing BOP between two ECG groups

ECG	Positive BOP N (%)	Negative BOP N (%)	P BOP (+) between groups
Group A: St elevation	34.8	65.2	
Group B: St depression	53.8	58.3	0.22

**Table 2:** Comparing dental indices, CAL, PD and DMFT between two ECG groups

	Group A ST elevation (n=23)	Group B ST depression (n=13)	P (Group AVs Group B)
CAL	2.1± 1.7	4.2±2.3	0.003
PD	2.16±1.1	2.21±0.32	0.84
DMFT	19.3±5.8	22.3±2.3	0.18

**Figure 3:**
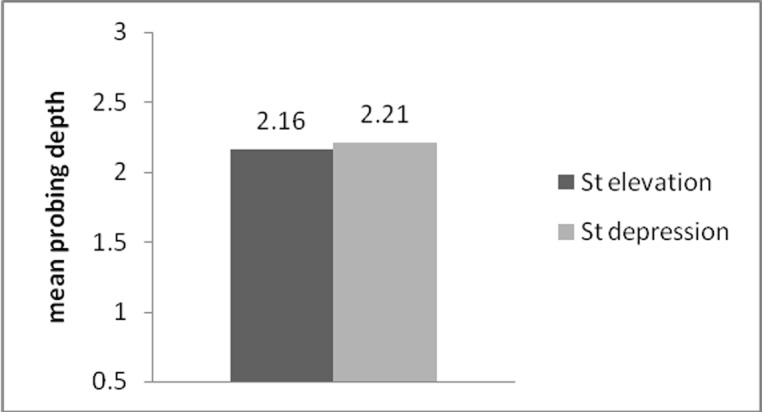
The difference between mean probing depth in two ECG groups was not significant (P=0.84).

Mean probing depth in group A was (2.16±1.1) and (2.21±0.32) in group B. No statistically significant differences were observed between probing depths between the two groups (P=0.84) (Table 2, Figure 3).

**Figure 4:**
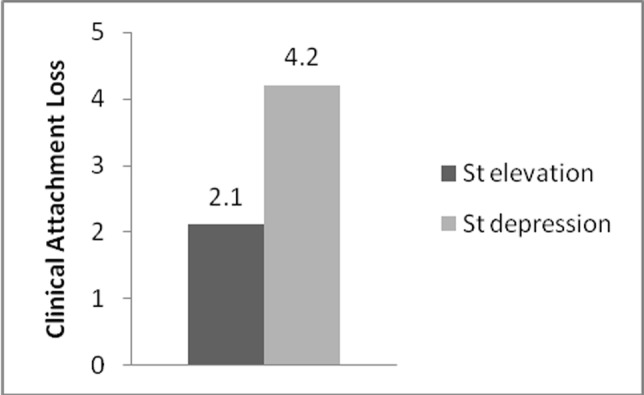
The difference in CAL between two groups was significant (P=0.003).

Clinical attachment loss was 2.1±1.7 in group A and 4.2±2.3 mm in group B (Table 2). As it has been shown in Figure 4 differences in CAL between the two groups were significant (P=0.003, OR=1.68) .

**Figure 5:**
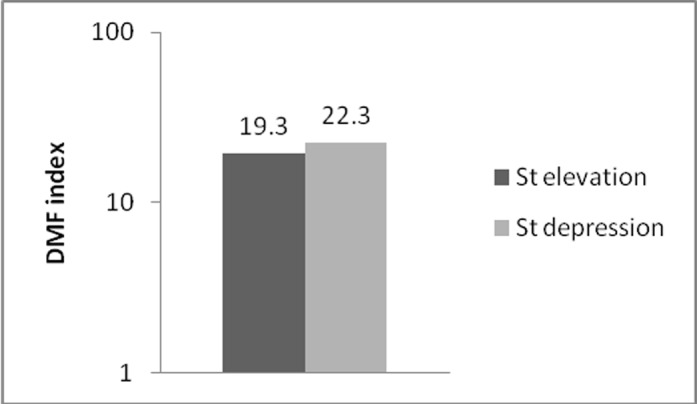
The difference in DMFT index between two groups was not significant (P=0.18).

DMFT index was 19.3±5.8 for group A and 22.3±2.3 for group B (Table 2). As shown in Figure 5, no significant differences were seen between DMFT index between groups A and B (P=0.18). Interactions between BOP, CAL, DMFT and PD were analyzed and no correlations were observed.

## Discussion

Oral and dental diseases are among the most prevalent diseases worldwide.Although these diseases are not important causes of mortality, they might have serious impact on the general health.^10^ Periodontal disease and dental caries are among the most common oral diseases.^11^ Streptococcus bacteria which are abundant in the oral cavity and are responsible for tooth caries can induce the platelets to bind fibrinogen, aggregate and eventually form a thrombus or platelet clot. In vitro studies have also shown that Porphyromonas gingivalis can induce the aggregation of human platelets.^2^ Although Birang et al did not reveal significant differences in coagulation factors between patients with gingivitis and a control group.^12^

Periodontitis is characterized by repeated episodes of inflammation that result from gram-negative bacteria. These bacteria have lipopolysaccharides (LPS) in their cell walls which activate the production of inflammatory mediators such as TNF-α and IL-1β. IL-1β itself is a promoter of smooth muscle cell proliferation and can be responsible for thickening of blood vessels walls.^2,13^

In 2005 Kosarvo et al proved the presence of two oral pathogens: Porphyromonas gingivalis and Actinobacillus actinomycetemcomitans, within the atherosclerotic plaque.^14^

Cueto et al revealed an association between periodontitis and acute myocardial infarction.^15^ However, Hujoel et al reported that such a correlation between coronary heart diseases and periodontitis does not exist.^16^ Joshipura et al believe that the controversies may relate to the fact that risk factors are different among different countries and nations.^17^ Several epidemiologic studies have been performed in Iran, which show that there is an association between oral health and cardiovascular diseases.^18-24^

Researchers have performed several studies on factors that might have a role in determining the prognosis of myocardial infarction. As Adler et al suggest ST segment elevation can be a predictive factor for poor outcome in patients with myocardial infarction.^8^ Okin et al showed that high levels of C-reactive protein in patients with ST segment depression is also predictive of a worst outcome.^25^ Barabess et al believe ST segment depression alone might be a prognostic feature in AMI patients without ST elevation.^9^ After studying the relation between microalbuminuria and major ECG changes, Tazeen et al^26^ showed that microalbuminuria in Asian populations can be a predictor for cardiovascular disease. In 2004, Tamaki et al^27^ investigated the association between periodontal conditions and electrocardiogram abnormalities and found no correlations between oral factors and the prevalence of electrocardiogram abnormalities. In the present study PD, BOP and DMFT indices were not significantly different between the study groups. CAL was significantly different between groups. In a study by Arabi et al,^18^ CAL was higher in patients with positive heart scan in contrast to control group with negative heart scan. They believe severe periodontal diseases would be expected to be seen in patients with positive heart perfusion scan.Although ST elevation is related to poorer prognosis in comparison to ST depression, as well as CAL, other indices were also all higher, even though not statistically significant, in the ST segment depression group. According to the results of the present study oral health status might be worse in patients with ST segment depression. Similar studies and studies investigating the relation between oral health and levels of ST segment depression are strongly recommended.

## Conclusion

No correlation was seen between probing depth, bleeding on probing and DMFT indices with ST segment changes. Clinical attachment loss was significantly higher in patients with ST segment depression.

## Acknowledgments

The authors would like to thank Dr. F Sadoughi for his kind cooperation in electrocardiogram interpretation.
